# Resolving "worm wars": An extended comparison review of findings from key economics and epidemiological studies

**DOI:** 10.1371/journal.pntd.0006940

**Published:** 2019-03-07

**Authors:** Muhammad Farhan Majid, Su Jin Kang, Peter J. Hotez

**Affiliations:** 1 Center for Health and Biosciences, James A Baker III Institute for Public Policy, Rice University, Houston, Texas, United States of America; 2 Department of Pediatrics, National School of Tropical Medicine, Baylor College of Medicine, Houston, Texas, United States of America; 3 Department of Biology, Baylor University, Waco, Texas, United States of America; Rollins School of Public Health, UNITED STATES

## Background

The major human helminthiases, including schistosomiasis and the soil-transmitted helminth (STH) infections, represent the leading neglected tropical diseases (NTDs) in terms of their global prevalence and ability to inflict tremendous disease burdens and disability [1]. Recent estimates from the Global Burden of Disease Study 2017 indicate that almost 1 billion people are infected with STHs, whereas 140 million people have schistosomiasis, with most living in the world’s low- and middle-income countries [2]. In response to the adverse health, educational, and economic effects of pediatric helminth infections, the World Health Organization (WHO) and other international development organizations have taken an active role in trying to lower the disease burden and improve child health through the use of preventive chemotherapy (PC), with a goal of reaching at least 75% of at-risk population and up to 100% of school-aged children [3–4]. By 2015, nearly 1 billion PC treatments were delivered by NTD PC programs globally [5].

Although the benefits of deworming programs for children have been known since the early 20th century [6–7], the modern framework for global deworming began with transmission modeling studies conducted by Anderson and May during the 1980s [8], which identified school-aged populations at greatest risk for heavy worm burdens, followed by work led by Bundy, Savioli, and others who provided proof-of-concept of the benefits of deworming through schools [9–10]. Later, the economists Miguel and Kremer conducted a key study in 2004 [11], which analyzed a randomized experiment conducted in Kenya in the late 1990s, and the results showed the significant effects of PC to improve child health and school attendance. More importantly, the effect was observed not only for the dewormed children but also other children in neighboring schools who did not directly get the treatments. Along with the “spillover” effect, authors suggested that PC is not only effective but highly cost-effective with a single treatment costing a few pennies. In many countries, deworming for STH infections and schistosomiasis has since been integrated with other mass treatment approaches that target lymphatic filariasis, onchocerciasis, trachoma, yaws, and other NTDs [12].

"Since the original findings highlighted above, multiple studies have confirmed that PC for STH infections and schistosomiasis leads to reduced worm burden [13–16], improved public health and DALYS [17–20] and high economic returns [21–23]. However, in 2012, and again in 2015, a Cochrane review found little to no evidence for the benefits of PC for STH on nutritional indicators, hemoglobin, and school performance [24–25]. Subsequently, a group of epidemiologists and researchers replicated and reanalyzed the original Miguel and Kremer study. The authors argued that there is little to no evidence for the benefits of PC on externalities [26–27], studies which were confirmed by an additional systematic review [28]. Yet another analysis argued that studies claiming the benefits of PC showed methodological biases [29]. The media quickly picked up on this issue [30–32], and the deworming/PC debate became a heated debate in the scientific community. The key study findings since 2000s by economists and epidemiologists that support and do not support PC are summarized in S1 Table. In this paper, we critically evaluate the “worm wars” literature, highlighting gaps in the current discourse on deworming, which have been ignored by both economists and epidemiologists. For the rest of this article, we mostly use the term mass drug administration (MDA) instead of PC because the former is the more commonly used term in the debates.

## Worm wars debate

A key question in the debate is whether school-based MDA in endemic areas is the preferred policy relative to individually testing and treating individuals. WHO currently supports MDA, and the following is the summary of the WHO recommendation based on the latest website update on Feb 20, 2018 [33]:

“Preventive chemotherapy (deworming), using annual or biannual^A^ single-dose albendazole (400 mg) or mebendazole (500 mg)^B^ is recommended as a public health intervention for all young children 12–23 months of age, preschool children 1–4 years of age, and school-age children 5–12 years of age (in some settings up to 14 years of age) living in areas where the baseline prevalence of any soil-transmitted infection is 20% or more among children, in order to reduce the worm burden of soil-transmitted helminth infection.

    A. Biannual administration is recommended where the baseline prevalence is more than 50%.

    B. A half-dose of albendazole (i.e., 200 mg) is recommended for children younger than 24 months of age.”

For Schistosomiasis, targeted distribution of praziquantel is the norm. Intervention frequency is determined by the prevalence of infection or of visible haematuria (for *S*. *haematobium* only) among school-age children.

But in their Cochrane review, Talyor-Robinson and colleagues stated, “The recommended drugs are effective at eliminating or greatly reducing worm infections, but the question remains whether doing so will reduce anaemia and improve growth, and consequently improve school attendance, school performance, and economic development, as has been claimed” [25]. Ultimately, we find some ambiguity in the debate around the following questions.

## Is the debate about all types of deworming or some types only?

At least six different helminths resulting in high prevalence human infections (in which more than 100 million people are infected) are currently targeted by deworming: the roundworm, *Ascaris lumbricoides*; the whipworm, *Trichuris trichiura*; two hookworms, *Necator americanus* and *Ancylostoma duodenale*; and two schistosomes, *Schistosoma haematobium* and *Schistosoma mansoni*. The major deworming drugs have differential effects on these helminths, especially when used in a single dose. Therefore, single-dose mebendazole (500 mg) is highly effective against *Ascaris* but not against the hookworms or *Trichuris* [34–36]. Single-dose albendazole (400 mg) is also highly effective against *Ascaris* but not *Trichuris* [34], and its effects against hookworm can be variable [37]. A one-time dose of praziquantel, which is typically administered on the basis of a child’s weight or height, is also highly effective against the two major human schistosomes. This information is important because the effects of deworming may vary depending on the major STH infection present in a community. For example, if *Trichuris* or hookworm is the predominant STH, then deworming with annual single-dose mebendazole may not produce major effects, compared to an area, say, where *Ascaris* is the predominant STH infection. This finding might explain why the original paper by Miguel and Kremer finds that deworming through single-dose albendazole had little effects on infection rates for whipworms [11]. As a result, some investigators have called for the development of better anthelminthic drugs or vaccines [38].

Along the same lines, it’s interesting to note that the Cochrane and other systematic reviews do not carefully differentiate STH species, which may explain why some interventions with single-dose albendazole or mebendazole appear to be ineffective.

The Miguel and Kremer study [11] and a systematic review indicate that the impact STH deworming drugs were more effective when used alongside praziquantel for schistosomiasis co-infections [39], whereas in another systematic review, schistosomiasis was found to be associated with learning, memory, and educational deficits [40], and praziquantel in two Cochrane reviews was shown to be effective for treating urogenital schistosomiasis caused by *S*. *haematobium* [41] and intestinal schistosomiasis caused by *S*. *mansoni* [42], respectively.

According to Miguel and Kremer:

“To summarize, treatment of schistosomiasis appears to be an extremely cost-effective health intervention under standard health cost effectiveness criteria for less developed countries, although this is less true for the treatment of geohelminths alone. Even in areas with geohelminths but little schistosomiasis, however, deworming is a cost-effective way to boost school participation relative to other educational interventions evaluated in the same area, such as directly reducing the cost of schooling through the provision of school uniforms. It also appears likely that deworming can be justified as a human capital investment” [11].

According to Pabalan and colleagues:

“Further, in Schistosoma infection co-prevalent settings, associations were generally stronger and statistically robust for STH-related deficits in learning, memory and reaction time tests (SMD:-0.36 to -0.55, P = 0.003–0.02).” [39].

Even Taylor-Robinson seems to agree about health benefits of treating populations with schistosomiasis. In their own words:

“The evidence for the benefit of treating populations with schistosomiasis is fairly clear [43], as the infection has a very substantive effect on health. However, this does not mean that a different drug treating a different helminth species is equally effective."

However, the evidence for the benefits of treating schistosomiasis simultaneously with STH infections do not approach the main question about MDA specifically targeting STH infections. This discrepancy may be resolved from the differential effects of mebendazole and albendazole, depending on the major STH species, as highlighted above, or by combining either albendazole or mebendazole with a second anthelminthic drug, such as ivermectin or oxantel pamoate to improve cure rates from deworming, or potentially newer generation anthelminthic drugs [38, 44–45].

## High intensity versus low intensity infections

Beyond helminth prevalence is the need to control for helminth intensity. For STH infections and schistosomiasis, the extent of end-organ pathologies, as well as effects on growth and cognition, are proportional to not only the presence of worms but also their presence in large numbers [8, 46]. Generally speaking, only moderate and heavy intensity helminth infections result in the major morbidities ascribed to pediatric helminth infections, such as anemia and other nutritional deficiencies, or physical growth stunting and cognitive deficits. Similarly, one can expect to see improvements in these pathologies only in the case of moderate and heavy infections, so that treatment of light infections would not be expected to show any benefits. We do recognize that low-intensity infections can result in significant pathology under some circumstances, such as in already extremely malnourished individuals or during pregnancy, but generally speaking, low-intensity worm infections would not result in obvious impairments that would improve following deworming. Our understanding is that the Cochrane analyses include studies with low worm intensities. The comparisons between the Miguel and Kremer study and the Cochrane and other systematic reviews need to be reevaluated through this lens.

## Life course: Ambiguities over choice of outcomes of study and conceptual frameworks

Current work offers no clear discussion of critical periods of physical growth and cognitive development across the life course. For instance, height for age—a measure of long-term development—is largely determined during early childhood with periods of catch-up growth in adolescence [47]. It is unclear why we should expect height for age to significantly improve in response to deworming in the first case between ages 6 and 10 [48]. Instead, there is increasing evidence for the benefits of deworming preschool age children in regards to reduced stunting, as well as reduced anemia in Africa [49], although an alternative meta-analysis failed to show benefits in children under five, in terms of mortality and growth [50]. A recent study on the cognitive effects of STHs, indeed, found that they may be maximal during early childhood development, possibly as young as the second year of life [51]. Therefore, deworming of young children, including toddlers (12 to 36 months) may offer the greatest benefits. In general, future research needs to pay attention to timing of deworming interventions with respect to the sensitive and critical periods in formation of developmental outcomes for children. Furthermore, realizing the full benefits of deworming may require long-term follow-up.

At the other end of the life spectrum, there is also interest in examining community-based deworming to include adults, with important collateral benefits on reducing the prevalence in children [52]. Specifically regarding school-age children, there is evidence to suggest that education has a positive causal effect on health and health behaviors. If true, the MDA-targeted school-aged population may be self-selected and better off compared to those school-aged children not at school. According to the United Nations Educational, Statistic and Cultural Organization (UNESCO) data in 2016, 63 million primary school-age children were out of school, and 61 million of lower secondary school-age children were out of school [53]. As a result, even if deworming can reduce absenteeism among children who enroll in schools, it is unclear whether deworming can be effective for those school-age children who are out of school. Additionally, Miguel and Kremer heavily emphasize the finding on schooling absenteeism [11]. The education literature often highlights the effects on years of schooling based on the highest grades attended, rather than how many years one has been in or out of school. This is because one may spend more years in school by repeating grades more often than doing well enough to advance to next grades. Baird and colleagues, in fact, show evidence that the increase in grade repetitions has a positive effect on secondary school attendance. It is unclear why overall grades attained show null effects, although some differences across gender were noted. [54].

## Is the debate really about net benefits of MDA versus individual testing?

Seen one way, the debate really is not about MDA or individual testing. If an area is populated enough with 99% infection, it would be hard to justify individual testing even by the Cochrane group, whereas if an area has just 1% infection and is populated enough, even Miguel and Kremer would find it hard to justify MDA. Is a 20% baseline infection rate the right threshold for annual deworming, with 50% the threshold for biannual deworming? Perhaps we should ask the following question instead: What is the optimal level of baseline infection (and levels of worm intensity) that can justify MDA rather than individual testing? Research and reviews that can map out the net benefits of MDA in almost continuous fashion from 0% to 100% baseline infection based on different geographic areas can perhaps help us move the debate forward in a more productive direction. However, it has been pointed out by several investigators that the costs of individual testing are not trivial and typically exceed by several-fold the actual costs of deworming [25, 55]. Further information on pairing baseline prevalence rates with intensity estimates would also be useful. Our point is to have an empirically driven and regionally informed threshold (with respect to both prevalence and intensity) to determine when MDA is required.

## Need to pay greater attention to global analysis of deworming

A big concern with existing work, however, is with inference or external validity, because the most influential work comes from East Africa with some studies from India and China not finding any serious externality effect of deworming [24–25, 56–57]. Recent studies are among the first to examine associations between worms and education/human development index (HDI) at the global level [58–60]. For instance, the study by Kang and colleagues found a negative but nonlinear effect of worms, which included steep effects in countries with low or modest worm prevalence levels (“worm indices”) but milder effects on countries with greater prevalence levels [60]. This work needs to be carefully built upon to better understand causal impact of deworming drugs in different parts of the countries with the varying level of the worm index to better understand the relationship between baseline prevalence and intensity of worms and treatment not only within countries but across the globe.

## Conclusions

STH infections and schistosomiasis represent some of the most common afflictions of childhood. WHO recommends PC/MDA as a low-cost approach to helminth control, and (given the excellent safety profile of anthelminthic drugs) there are few serious side effects of deworming drugs. There is evidence to support the WHO policy [61]. Yet others have questioned the rationale for MDA, arguing that the benefits of MDA are smaller than its costs, or even exaggerated [24–25]. In this paper, we critically evaluate the state of “worm wars.” Our reading of the literature reveals the following key insights.

1. The debate is not about all types of worms. The primary debate is about the effectiveness of MDA against STH infections rather than schistosomiasis.
2. Even among the STH infections, there are marked differences in their susceptibility to single-dose anthelminthic drugs. Such findings have led to calls to either supplement deworming programs with additional anthelminthic drugs, such as ivermectin or oxantel, or to develop new chemical entities and even anthelminthic vaccines [62]. Ultimately, the published intervention studies need to be reanalyzed with this anthelminthic-specific lens, as well as the need to differentiate studies looking at light versus heavy intensity infections.
3. In some areas, STHs may exert their greatest cognitive and physical growth effects on preschool-age children, and even toddlers. Some of the newer findings on this cohort might require reconsideration for our current approaches to deworming. At the same time, deworming adult populations may produce greater benefits for the entire community, including children. Incorporation of life histories should become an important component of the dialogue around helminth control and elimination.
4. Beyond the changes in anthelminthic regimen, another debate is not about whether policymakers need to individually test and then give deworming treatments or conduct MDA, but it's about the optimal threshold at which MDA should be recommended. Currently WHO recommends MDA for places with 20% or more STH infection prevalence rates. The debate could shift in terms of what threshold should be applied in different contexts based on the evidence of the cost-effectiveness of deworming in those contexts. Yet, currently, the literature does not see the debate this way and is stuck on a binary vision of supporting MDA or not. Research should highlight the effectiveness of MDA for communities with different baseline worm infections rates (potentially both for prevalence and intensity) in different contexts to help us better understand the optimal threshold.
5. More research is needed on the external validity of findings from East Africa versus other global areas where helminth infections are widespread, with the possibility that we might see regional variation depending on baseline human development and economic indices.

Convening experts along these lines might help to resolve a number of the current disputes around PC/MDA.

## Supporting information

S1 Table Summary of key studies of mass deworming. (DOCX)
