# Supplementary material for: Resolving "worm wars": An extended comparison review of findings from key economics and epidemiological studies
Source: PLoS Negl Trop Dis. 2019 Mar 7;13(3):e0006940. doi: 10.1371/journal.pntd.0006940 (PMC6405048; doi:10.1371/journal.pntd.0006940)
Supplement: S1 Table — (DOCX) [file pntd.0006940.s001.docx]

| **Studies largely supporting MDA** | | **Studies largely against MDA** | |
| --- | --- | --- | --- |
| **Title and citation** | **Reported evidence and claims** | **Title and citation** | **Reported evidence and claims** |
| **Studies published between 2000- 2010** | | | |
| Worms: Identifying impacts on education and health in the presence of treatment externalities (Miguel and Kremer, 2004) [11] | The authors studied a randomized school-based deworming program in rural western Kenya from 1998 through 1999, where students received albendazole twice a year; in addition, some schools received praziquantel for schistosomiasis infections annually. They found large reductions in worm infections among treated children and children in schools located near treatment schools. They estimated an 18% point reduction after one year in the proportion of moderate-to-heavy infections among untreated individuals attending treatment schools and a 22% point reduction among individuals attending a school within 3km of a treatment school. | Effects of treatment for intestinal helminth infection on growth and cognitive performance in children: systematic review of randomised trials (Dickson et al., 2000) [66] | Dickson and colleagues did a systematic review of 30 randomized controlled trials to investigate the effects of deworming (anthelmintic drug) treatment on growth and cognitive performance of more than 15,000 children between 1- 16 years of age. The results of one to multiple dose administration showed that there was increase in weight gain (0.24-0.38kg for one dose and 0.10-0.12kg for multiple dose). However, these results were not consistent between trials, and there was no significant/ sufficient evidence on improvement of cognitive performance. |
| Effect on weight gain of routinely giving albendazole to preschool children during child health days in Uganda: cluster randomised controlled trial (Alderman et al., 2006) [63] | Alderman and colleagues explored a program delivered by community-based organizations during 2000-2003 across 48 parishes in 5 districts in eastern Uganda. This program area was also characterized by high worm prevalence, with an infection rate of over 60 percent in children aged between 5-10 years old. Using a cluster-randomization approach, parishes were randomly assigned to receive either the standard intervention. Their findings showed that when mass treatment was given twice a year, child weight increased about 10% whereas annual treatment led 5% weight gain. | Anthelmintic treatment of rural Bangladeshi children: effect on host physiology, growth, and biochemical status (Northrop-Clewes et al., 2001) [67] | This research was the first longitudinal study that assessed the benefits of regular deworming treatment (mebendazole) on growth, physiology, and biochemical status in children. They conducted a 12 months of longitudinal intervention (at 2, 8, and 12 months) for 123 children in Bangladesh aged 2-5 years. The results showed no significant difference in the growth and nutritional status improvement of treated children compared to the untreated. The authors questioned about the effect of deworming but noted that the lack of improvement could be due to several factors such as a low prevalence and intensity of helminth infection in children in a poor area, sample size, etc. |
| Anemia and School Participation (Bobonis, Miguel, and Puri-Sharma, 2006) [22] | The authors evaluated the impact of a randomized health intervention, delivering iron supplementation and deworming drugs to preschool children in India. At baseline, 69% were anemic and 30% had intestinal worm infections. The evidence showed weight gain among assisted children. Also, preschool-participation rates rose by 5.8 % points, reducing absenteeism by one-fifth. Gains were pronounced for those most likely to be anemic at baseline. Despite the acknowledgment of the unknown long-term effects of child health on adult income and life chances, the authors viewed school-based health programs are cost-effective to promote school participation. |  |  |
| Disease and Development: Evidence from Hookworm Eradication in the American South (Bleakley, 2007) [23] | The author revisited the impact of hookworm eradication in the U.S. South, exploiting a program launched in 1910. To assess the impact of this intervention on educational and economic outcomes, Bleakley used data from the 1940 U.S. census to compare adult outcomes among birth cohorts who entered the labor force before and after the deworming campaign in the U.S. South. Those children, who were exposed to deworming in the past, were more likely to have higher earnings (43% increase in wages) as adults. This effect suggests that hookworm infections could have explained as much as 22% of the income gap between the U.S. North and South at the time. Given initial infection rates of 30%-40%, hookworm eradication would therefore imply a long-run income gain of 17% (based on 43% increase in wages and a 40% infection rate) |  |  |
| Effect of administration of intestinal anthelmintic drugs on haemoglobin: systematic review of randomised controlled trials (Gulani et al., 2007) [20] | The researchers did a systematic review which includes 14 randomized controlled trials with 7,829 subjects to assess the effect of routine administration of deworming (intestinal anthelmintic) drugs on haemoglobin level. The results showed a marginal increase in haemoglobin concentration (1.71g/l) which indicates a small reduction (5% to 10%) in the prevalence of anaemia in communities where prevalence of intestinal helminthiasis is relatively high. |  |  |
| **Studies published after 2010** | | | |
| Worms at work: long run impacts of a child health investment (Baird et al., 2016) [54] | The authors did a long-term follow-up on M&K [14]. Ten years after deworming treatment, men who were eligible as boys stay enrolled for more years of primary school, work 17% more hours each week, were more likely to hold manufacturing jobs, and missed one fewer meal per week. Women who were in treatment schools as girls were approximately one quarter more likely to have attended secondary school. They also reallocated time from agriculture into nonagricultural self-employment. The authors estimated a conservative annualized financial internal rate of return to deworming of 32% and showed that MDA may generate more in future government revenue than it costs in subsidies. | Deworming school children in developing countries (Taylor-Robinson et al., 2015) [25] | The researchers examined findings of 45 trials, including nine cluster-RCTs. Eight trials were in children known to be infected, and the other 37 trials were completed in endemic areas, including high (15 trials), moderate (12 trials), and low prevalence areas (10 trials). The analysis showed that treating the infected children had some nutritional benefits such as the increase in weight gain and haemoglobin (low quality evidence). However, they found little or no benefits on school attendance, cognitive functioning, or physical-wellbeing. Also, treating all children in endemic region showed little or no nutritional benefits which also differed across trials. |
| Does mass deworming affect child nutrition? Meta-analysis, cost-effectiveness, and statistical power (Croke et al., 2016) [64] | The researchers augmented Cochrane review sample [21] as well as several excluded studies and then conducted meta-analysis on this augmented sample. Focusing on weight gain, for which the number of available studies is greatest, they noted that the appropriate test for the hypothesis of no treatment effect in all cases is a fixed-effect meta-analysis. Using this model, the hypothesis of zero weight gain from deworming was rejected at the 10% level using the original data from the Cochrane review study. Using the augmented sample, they found a 0.111 kg weight gain (P < 0.001) from deworming in a fixed-effects model and a 0.134 kg weight gain (P = 0.01) in a random-effects model. | Re-analysis of health and educational impacts of a school-based deworming programme in western Kenya: a pure replication (Aiken et al., 2015) [26] | The researchers followed the original data and code from M&K [14]. While most of the original findings remained the same, some results such as anaemia reduction as well as improvement in nutrition and school performance showed little or no evidence. Moreover, after correcting coding errors in the original study, the notable “spillover” effect on neighboring schools disappeared in the re-analysis. The average effect in the 3km to 6km showed only 0.25 in which combining all effects suggested the effect is almost equal to zero. The authors concluded that “there was little evidence of an indirect [spillover] effect on school attendance among children in schools close to intervention schools.” |
| Differential effect of mass deworming and targeted deworming for soil-transmitted helminth control in children: a systematic review and meta-analysis (Clarke et al., 2017) [52] | The study did a systematic review and meta-analysis of empirical evidence on the targeted school-aged children and the effect of community-wide mass deworming. Among 56 studies, all of which were used for the systematic review, 38 of them were used for meta-analysis. They utilized inverse variance weighted generalized linear models, with prevalence reduction to examine the effect of mass versus targeted drug administration. Although this study did not look at the external benefits of deworming, however, the study found round treatment of adult would have indirect benefits on reducing the prevalence of soil-transmitted helminths in the high-risk group of school-aged children. | Re-analysis of health and education impacts of school-based deworming programme in western kenya: a statistical replication of cluster quasi-randomized stepped-wedge trial (Davey et al., 2015) [27] | The authors re-analyzed the M&K study [14], but they utilized different statistical methods, using cluster quasi-randomized stepped-wedge trial on two outcomes: school attendance and achievement. They addressed a significant amount of missing data found on the year-stratified cluster-summary analysis. Next, there was no clear evidence observed either on school attendance or performance. On year-stratified regression model, they found some evidence of improvement in school attendance, but no evidence on school performance. Finally, when both years combined, strong evidence was found on improvement in school attendance only but no school performance. |
| Exploiting Externalities to Estimate the Long-Term Effects of Early Childhood Deworming (Ozier, 2018) [65] | The author studied the same randomized program in Kenya but focused on children who were ages 0-2 years who were not directly treated to investigate benefits from positive within-community externalities generated by the mass school-based deworming. After 10 years of the program, the study found a large cognitive effect which is comparable between 0.5 -0.8 years of schooling for these individuals but no evidence on height or stunting. The findings suggested that these children benefited primarily through the reduced transmission of worm infections and the effects were twice as large among children with an older sibling in the participated school. | The impact of mass deworming programmes on schooling and economic development: an appraisal of long-term studies (Jullien, Sinclair, and Garner 2016) [29] | The researchers evaluated all long-term follow-up studies from Croke [55], Baird [46], and Ozier [59] which have more than 9 years after cluster-randomized trials in Kenya and Uganda. The results showed the conflicted findings of deworming (soil-transmitted helminths) in low- and middle-income countries. Also, despite clear evidence presented in the previous studies, they found a number of substantial methodological bias and problems in reporting in the 11 reports in them. The authors addressed specific issues found in each study as well as the absence of cumulative effects from multiple rounds of deworming, pre-planned protocols and post hoc subgroup analyses observed in overall deworming studies. |
| Deworming in pre-school age children: A global empirical analysis of health outcomes (Lo et al., 2018) [49] | Lo and colleagues assessed the association between deworming in pre-school age children (1-4 years old) and specific health outcomes (underweight, stunting, and anemia). They utilized nationally representative cross-sectional, individual-level survey data conducted in 45 STH endemic countries with 325,115 children between 2005- 2016. The evidence showed that those treated children were less likely to be stunted (1.2 % point decline from mean of 26%) and less likely to have anemia (1.8% point decline from mean of 58%) but showed no consistent effect on weight gain. Based on the findings drawn from the individual-level data, the authors recommend continual deworming for pre-school age children. | Effects of deworming on child and maternal health: a literature review and meta-analysis. (Thayer et al., 2017) [50] | The authors reviewed literature on outcomes of mass deworming for children 5 years old or less (27 trials) and pregnant women (11 trials). They specifically focused on whether current evidence of deworming can be included as an intervention in the Lives Saved Tool (LiST), a software package estimating the effect of interventions on maternal and child health outcomes. The findings showed that the effects of deworming on mortality, anemia, or growth were not consistent in children younger than five as well as women of reproductive age. In other words, they found no population level effect. Based on the results, the authors suggested deworming should not be included as an intervention in the LiST model. |
| The public health benefit and burden of mass drug administration programs in Vietnamese schoolchildren: Impact of mebendazole (Debaveye et al., 2018) [17] | The study assessed the health benefit of MDA (mebendazole) in eight million Vietnamese children aged 5-14 years between 2006-2011 to compare it to the public health burden of the pharmaceutical supply chain and resource use. They used a Markov model which predicts the prevalence of soil transmitted helminthiasis and was combined with disability weights to calculate the population morbidity. Also, the public health burden of the pharmaceutical supply chain of mebendazole was calculated. The results suggested that the public health benefit of deworming is 18,035 times larger than the public health burden of pharmaceutical production. The authors viewed the cost-effusiveness is not limited to the monetary costs but also to the environmental impacts such as decline of emissions. | Mass deworming to improve developmental health and wellbeing of children in low-income and middle-income countries: a systematic review and network meta-analysis (Welch et al., 2017) [28] | The authors assessed the effect of mass deworming for soil-transmitted helminths and schistosomiasis separately on growth, school attendance, cognition, performance, and quality of life in children in endemic areas. They examined 52 studies of nearly 1.3 million children and four long-term trials with more than 160,000 children, conducted 8-10 years after MDA was initiated. Their aggregate level data included randomized and quasi-randomized trials, controlled before-after studies, interrupted time series. They also controlled numerous factors as a baseline consideration such as nutritional status, different types of worm infection, micronutrients, learning environments, etc. The findings revealed no “spillover” effect for the untreated children in the community. Also, the overall evidence of deworming on cognition, literacy, and school enrollment were inconsistent and not significant. |

**Additional References (in supplemental table only):**

1. Alderman H, Konde-Lule J, Sebuliba I, Bundy D, Hall A. Effect on weight gain of routinely giving albendazole to preschool children during child health days in Uganda: cluster randomised controlled trial. BMJ. 2006; 333(7559):122.
2. Croke K, Hicks JH, Hsu E, Kremer M, Miguel E. Does Mass Deworming Affect Child Nutrition? Meta-analysis, Cost-Effectiveness, and Statistical Power. 2016. Report No.: 22382. Available from: <http://www.nber.org/papers/w22382>. [cited 11 June 2018].
3. Ozier O. Exploiting Externalities to Estimate the Long-Term Effects of Early Childhood Deworming. American Economic Journal: Applied Economics. 2018; 10(3):235–62.
4. Dickson R, Awasthi S, Williamson P, Demellweek C, Garner P. Effects of treatment for intestinal helminth infection on growth and cognitive performance in children: systematic review of randomised trials. BMJ. 2000; 320: 1697–1701.
5. Northrop-Clewes CA, Rousham EK, Mascie-Taylor CN, Lunn PG. Anthelmintic treatment of rural Bangladeshi children: effect on host physiology, growth, and biochemical status. Am J Clin Nutr. 2001; 73(1):53–60.
